# Assessing the diagnostic, prognostic, and therapeutic potential of the somatostatin/cortistatin system in glioblastoma

**DOI:** 10.1007/s00018-025-05687-9

**Published:** 2025-04-23

**Authors:** Miguel E. G-García, Ana S. De la Rosa-Herencia, Álvaro Flores-Martínez, María Ortega-Bellido, Rafael Sánchez-Sánchez, Cristóbal Blanco-Acevedo, Manuel D. Gahete, Juan Solivera, Raúl M. Luque, Antonio C. Fuentes-Fayos

**Affiliations:** 1 Department of Cell Biology, Physiology, and Immunology, University of Cordoba, 14014, Cordoba, Spain / Maimonides Biomedical Research Institute of Cordoba (IMIBIC), 14004, Cordoba, Spain / Reina Sofia University Hospital (HURS), 14004, Cordoba, Spain; 2 Department of Cell Biology, Physiology, and Immunology, University of Cordoba, 14014, Cordoba, Spain; 3 Maimonides Biomedical Research Institute of Cordoba (IMIBIC), 14004, Cordoba, Spain; 4 Reina Sofia University Hospital (HURS), 14004, Cordoba, Spain; 5 Pathology Service, Reina Sofia University Hospital, 14004 Cordoba, Spain; 6 Department of Neurosurgery, Reina Sofia University Hospital, 14004 Cordoba, Spain; 7 Board of Governors Regenerative Medicine Institute, Cedars-Sinai Medical Center, Los Angeles, CA 90048 USA; 8 CIBER Physiopathology of Obesity and Nutrition (CIBERobn), 14004, Cordoba, Spain

**Keywords:** Somatostatin receptor subtype 1, Somatostatin receptor subtype 2, Glioblastoma, Diagnostic/prognostic biomarkers, Therapeutic targets, Somatostatin analogs

## Abstract

Glioblastoma remains an incurable tumour (median survival: ~ 15 months) and little clinical progress has been made over the past decades. Therefore, identification of novel biomarkers and therapeutic targets is imperative. Targeting the somatostatin/cortistatin-system is considered a successful avenue for treating different tumour pathologies. Thus, we comprehensively characterized (clinically and molecularly) the expression of the somatostatin/cortistatin-system components [ligands and receptors (SSTRs)] using five cohorts of patients and tested the *in-vitro* therapeutic response of different SSTR-agonists and somatostatin analogs (SSAs) in primary patient-derived glioblastoma cells. A clear downregulation of the whole somatostatin/cortistatin-system (except for SSTR5) in glioblastoma *vs*. non-tumour brain samples was demonstrated, with high discriminatory capacity. Moreover, poor overall-survival and critical aggressiveness-parameters (*i.e.,* recurrence, *IDH1*-wildtype and G-CIMP status, classical and mesenchymal GBM-subtypes, *EGFR*-amplification) were robustly associated with SSTR1/SSTR2 downregulation. Notably, octreotide, pasireotide, and SSTR1/2/5-agonists treatments significantly reduced cell-proliferation in primary patient-derived GBM-cells. Molecularly, antitumour effects of octreotide/pasireotide were exerted through key signalling-factors related to glioblastoma-aggressiveness (*i.e.,* CDKN1A-B/JAK-STAT/NF-κB/TGF-β-pathways). Altogether, this study demonstrated that somatostatin/cortistatin-system is drastically altered in GBM representing a useful prognostic tool, and that SSTR-modulators might represent a potential therapeutic strategy to treat specific subsets of patients with GBM.

**Supplementary Information:**

The online version contains supplementary material available at 10.1007/s00018-025-05687-9.

## Introduction

Glioblastoma (GBM) stands as the most common and lethal brain tumour in adults, constituting nearly half of all diagnosed cases [1, 2]. Unfortunately, it presents a hopeless five-year survival rate of less than 10% for newly diagnosed patients [3, 4]. GBM management involves maximal surgical resection followed by a combination of radiation and chemotherapy [5, 6]. Regrettably, there is currently a lack of effective pharmacological therapy for this devastating disease, with most FDA-approved treatments offering only marginal survival benefits [6]. Thus, there is an urgent need for novel biomarker approaches and therapeutic strategies.

Over the last years, an emerging approach in cancer treatment has been based on the repositioning of drugs already approved to treat other pathologies, since they can be easily used in clinical practice compared to new drugs [7, 8]. Herein, somatostatin (SST) and cortistatin (CORT) system [ligands and receptors (SSTR1-5) [9–11]) has been classically described as the gold-standard inhibitory system for cell growth (modulating apoptosis and proliferation [12]), which have been therapeutically evaluated and explored in several cancer types [13–24]. However, although some studies have evaluated the value of specific components of the SST/CORT-system in GBM [22, 25–31], the information included in these reports is quite limited, fragmentary and, in some cases, unclear. Therefore, our current understanding of the molecular profile of all the components of the SST/CORT-system, and their clinical and pharmacological relevance in GBM cells remains inconsistent and requires further investigation. Accordingly, the present study was conceived to characterize the molecular profile of SST/CORT ligands and SSTR1-5 in GBM tissues *vs*. non-tumour brain samples, to highlight their potential clinical associations with key clinical parameters of aggressiveness, and, to determine, for the first time, the potential clinical benefits that SST/CORT neuropeptides, synthetic SSAs (octreotide, lanreotide, and pasireotide), and SSTR agonists (ago-SSTR1, ago-SSTR2, ago-SSTR3, and ago-SSTR5) could exert in the proliferation of primary GBM cell cultures, and the signalling pathways/mechanisms that could be mediating the potential antitumour effects of SSAs.

## Materials and methods

### Sample collection

Fresh GBM samples (*n* = 62) were obtained after intracranial surgery, while non-tumour samples (*n* = 10; control samples) were obtained from healthy brain donors from the Reina Sofia University Hospital (Cordoba, Spain; autopsies and lobectomy surgeries in epileptic patients), as previously described [32]. All samples underwent histological confirmation by expert pathologists following the WHO 2016 classification. A portion was rapidly frozen in liquid nitrogen and stored at − 80 °C for subsequent total-RNA extraction (as described below). Simultaneously, another portion was fixed in formalin, embedded in paraffin (formalin-fixed paraffin embedded; FFPE) for immunohistochemical (IHC) analysis (as described below). Moreover, when possible, a fresh tissue sample was collected [from Reina Sofia University Hospital (Cordoba, Spain) and Virgen del Rocio University Hospital (Seville, Spain)] and introduced in cold S-MEM to carry out primary GBM cell-cultures (as described below). Patient´s demographic and clinical features were systematically recorded to perform clinical correlations (internal cohort: Table 1).

**Table 1:**
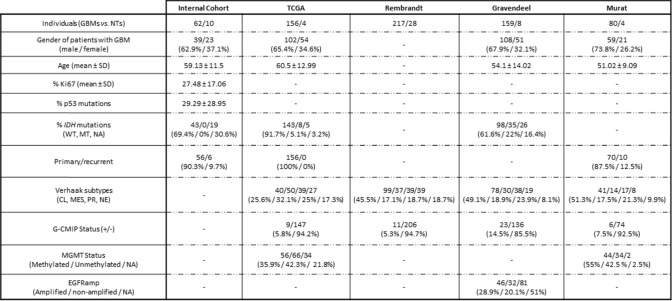
Demographic, clinical, and molecular data from an internal cohort and four external cohorts with GBM patients and non-tumour (control) individuals

Available information recollected in our **Internal Cohort** (*n* = 62 GBM samples *vs*. *n* = 10 Non-tumour samples), **TCGA Cohort** (*n* = 156 GBM samples *vs*. *n* = 4 Non-tumour samples), **Rembrandt Cohort** (*n* = 217 GBM samples *vs*. *n* = 28 Non-tumour samples), **Gravendeel Cohort** (*n* = 159 GBM samples *vs*. *n* = 8 Non-tumour samples), and **Murat Cohort** (*n* = 80 GBM samples *vs*. *n* = 4 Non-tumour samples).

### Data availability for external cohorts.

To corroborate the results obtained from our internal cohort, we employed GlioVis-Tools (http://gliovis.bioinfo.cnio.es) for analysing transcriptomics data from GBM patients. Specifically, the evaluated datasets include: (i) TCGA (bulk-RNAseq data; *n* = 156 GBM, *n* = 4 non-tumour) [33]; (ii) Rembrandt (microarray data; *n* = 219 GBM, *n* = 28 non-tumour) [34]; (iii) Gravendeel (microarray data; *n* = 159 GBM, *n* = 8 non-tumour) [35]; and (iv) Murat (microarray data; *n* = 80 GBM, *n* = 4 non-tumour) [36]. Moreover, we examined phenotype data from these external datasets to uncover relevant clinical correlations [*e.g*., *IDH*-status, recurrence, etc. (Table 1)]. Additionally, to determine the relevance of the SST/CORT-system in recurrent tumours, dataset from the Glioma Longitudinal AnalySiS (GLASS) consortium was downloaded from “https://glass-consortium.org/”. Single-cell RNAseq data of adult GBM were downloaded from Single-cell-Portal—Broad-Institute (GSE131928; total cells, *n* = 5528) and analysed using Seurat-packageV3. Filtering, normalization, and clustering were performed as described previously [37]. Signature score based on the mRNA expression of Neural Progenitor Cells (NPC) markers (*SOX2*, *NES*, and *ASCL1*) were performed using R package called “UCell”.

### RNA isolation, retrotranscription, and gene expression analyses.

Total-RNA was extracted from all samples, and concentration and purity were determined as previously reported [8, 32, 37]. Then, RNA (1 μg) was retrotranscribed to cDNA using random hexamer primers, as previously reported [8, 32, 37]. To determine the expression of the SST/CORT-system [ligands (*SST*/*CORT*) and receptors (*SSTR1*/*SSTR2*/*SSTR3*/*SSTR4*/*SSTR5*)] and three housekeeping genes [β-actin (*ACTB*), hypoxanthine–guanine phosphoribosyl-transferase (*HPRT*), glyceraldehyde 3-phosphate dehydrogenase (*GAPDH*)] simultaneously across all human samples (GBMs *vs*. non-tumours), a qPCR dynamic array system based on microfluidic technology (Fluidigm) was used using specific and validated primers (Table 2), as recently reported [8, 32, 37]. To control for variations in RNA-quantity and retrotranscription reaction efficiency, the copy number of each transcript was adjusted with a normalization-factor (NF) calculated from the three housekeeping genes and the GeNorm 3.3 software, as previously reported [8, 32, 37].

**Table 2:**
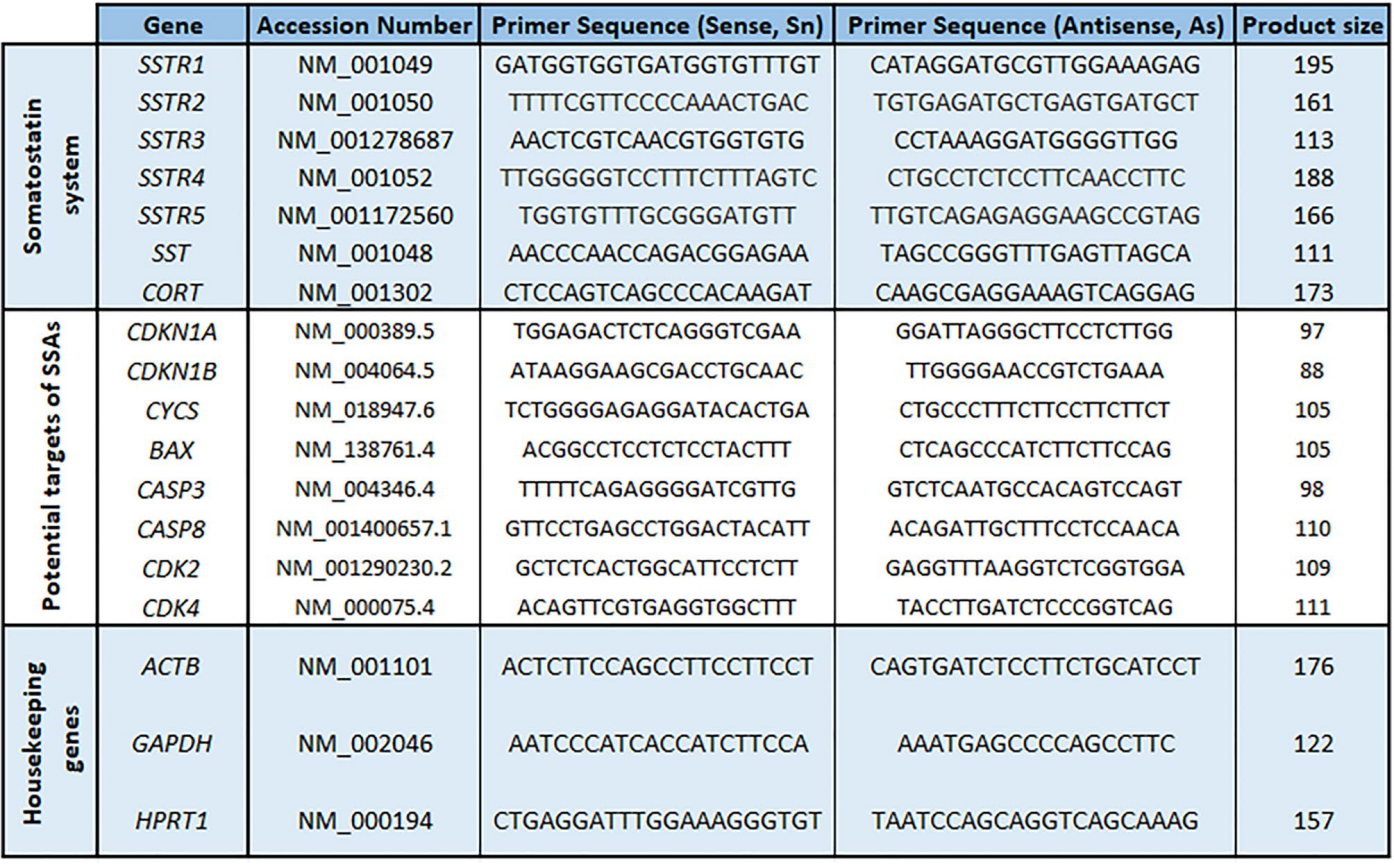
Specific primers for human transcripts used in this study [SST/CORT-system (*SSTR1-5*, *SST*, and *CORT*), cell-cycle/apoptotic regulators (*CDKN1*, *CDKN1B, CDK2, CDK4, CYCS, BAX, CASP3,* and *CASP8*), and **three Housekeeping Genes** (*ACTB*, *GAPDH*, and *HPRT1*)], were specifically designed and used in qPCR-based microfluidic and RT-qPCR assays

The official name of the genes, NCBI accession number of the transcripts, primers sequences (Sense and Antisense), and product sizes of the amplification products are included.

### SSTRs immunohistochemistry (IHC) analysis

A representative subset of tissue samples from our internal cohort (*n* = 13 GBM and *n* = 4 control samples) were utilized for IHC analysis following protocols previously described [32, 38]. Briefly, SSTR1 (clone UMB7; ab137083; Abcam) and SSTR2 (clone UMB1; ab134152, Abcam) monoclonal antibodies were used at 1/50 and 1/200, respectively. Antigen presence was detected using the UltraView Universal DAB Detection Kit (Ventana), and slides were immuno-stained simultaneously to mitigate inter-experiment variability. SSTRs stainings were independently analysed by two experienced pathologists using a double-blind approach. Staining intensity in GBM was classified qualitatively as negative [samples showing null or extremely low expression (compared to control tissues exceeding 95%), low (samples with a loss of intensity ranging between 80–95%), or positive (SSTR positive expression surpassing 80% of the total area)].

### Primary patient-derived GBM cell culture

Fresh tissue samples were immediately collected after intracranial surgery in sterile, cold S-MEM medium (complemented with 0.1% BSA, 0.01% l-glutamine, 1% antibiotic–antimycotic solution, and 2.5% HEPES) and dispersed into single cells using a mechanic/enzymatic protocol. Then, single-cells were cultured onto coating poly-L-Lysine tissue-culture plates in D-MEM-DV containing 10% FBS and complemented as S-MEM medium, as previously detailed [8, 32, 37]. Clinical information from these GBM patients is included in Table 3.

**Table 3:**
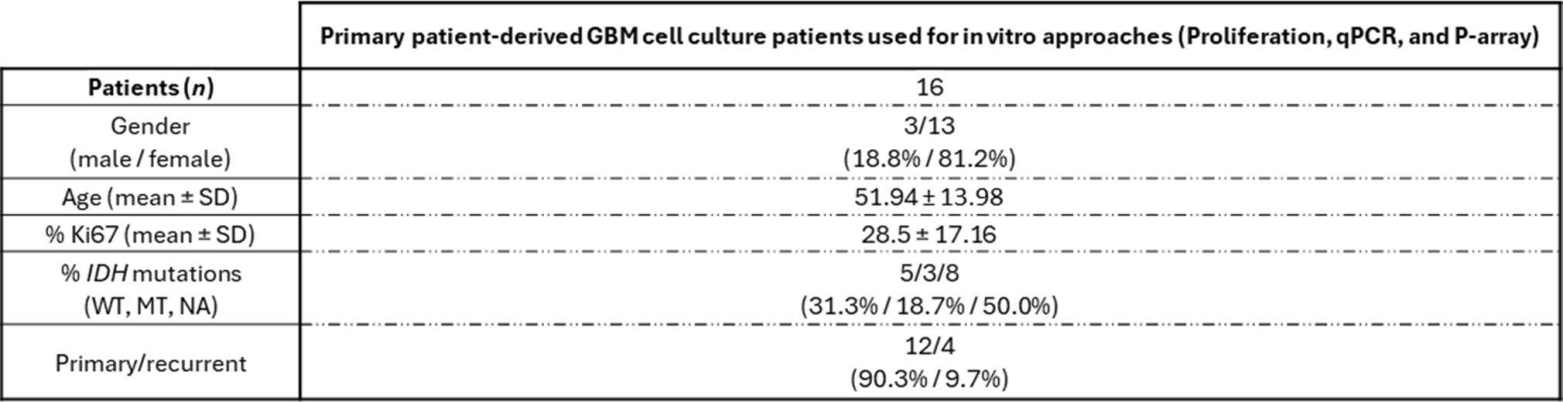
Demographic, clinical, and molecular information from GBM patients included in primary patient-derived cell cultures in vitro (Proliferation assay, qPCR, and Phospho-Array)

### Cell proliferation and apoptosis assays.

GBM cells were seeded in 96-well plates at the density of 10,000 cells/well (~ 75% cell confluence) and were serum-starved for 24 h. The assessment of proliferation/viability was assessed (every 24 h for up to 72 h) using the Resazurin reagent, following methods previously described [8, 32, 37] in response to: (i) different SSAs [first generation (octreotide and lanreotide) and second generation (pasireotide)], and (ii) different subtype-selective agonists for SSTRs [for SSTR1 (BIM-23926), SSTR2 (BIM-23120), SSTR3 (BIM-355), and SSTR5 (BIM-23206), generously provided by IPSEN Biomeasure (Milford, MA, USA); specificity of these human agonists was previously reported [13, 39, 40])] at a concentration of 10^−7^ M (dose previously reported to be the lowest that caused a maximal effect in different cell cultures models [13, 24, 40, 41]). Apoptosis rate was performed in primary GBM cells (40,000 cells/well onto white-walled multi-well luminometer plates) in response to octreotide and pasireotide (48 h of incubation) using Caspase-Glo® 3/7 Assay (Promega), as previously reported [8, 32, 37]. Results were expressed as a percentage relative to vehicle-treated controls.

### Human phosphorylation pathway profiling array

As previously reported [8, 30], the Human Phosphorylation Pathway Profiling Array C55 kit (#AAH-PPP-1-4, Raybiotech, Inc.) was used to analyse the differential phosphorylation levels of 55 proteins belonging to 5 key oncogenic pathways (MAPK, AKT, JAK/STAT, NF-κB, and TGF-β) in response to octreotide and pasireotide [pool of *n* = 3 independent experiments from primary patient-derived vehicle-treated *vs*. octreotide-treated or *vs*. pasireotide-treated GBM cells], following the manufacturer’s instructions. Densitometric analysis of the array spots was carried out with ImageJ software, making use of positive control spots as a normalizing factor.

### Statistical and bioinformatic analysis

Clustering, heatmaps, and Principal Component Analysis (PCA) analyses were performed with MetaboAnalyst Software v.5.0 (McGill University, Quebec, Canada). All data are presented as mean ± SEM. All statistical analyses, including ROC (Receiver Operating Characteristic) Curve analysis, were conducted using GraphPad Prism v.8.0 (San Diego, CA, USA). Normality was assessed using Shapiro or Kolmogorov–Smirnov test. Consequently, either parametric (Student t) or nonparametric (Mann–Whitney U) tests were implemented to analyse the relationships between risk factors, clinical data, and molecular profile of the components of the SST/CORT-system. For categorical variables, k‐square test was used. For survival analysis, groups were determined based on the cut-off points established by the survminer R package (R language v.4.1), and Log-Rank and Gehan–Wilcoxon tests were applied to compare Overall Survival according to expression levels of the SST/CORT-system components. In the Human Phosphorylation Array, a fold change (log2) of 0.2 was considered as a significant threshold, as previously reported [8, 30]. To generate JAK/STAT and NF-κB scores, Z-scores of genes included in KEGG_JAK_STAT_SIGNALING_PATHWAY (“Transcription factors”) and BIOCARTA_NFKB_PATHWAY were performed in available human external datasets. These set of genes were downloaded from GSEA webpage (“https://www.gsea-msigdb.org/gsea/index.jsp”). Statistical significance was set at (*) p ≤ 0.05, (**) p ≤ 0.01, (***) p ≤ 0.001, (****) p ≤ 0.0001, compared to control conditions. A trend for significance was indicated when p-values ranged between > 0.05 and < 0.1.

## Results

### Expression profile SST/CORT-system components is drastically downregulated in GBM vs. control samples

**Fig. 1:**
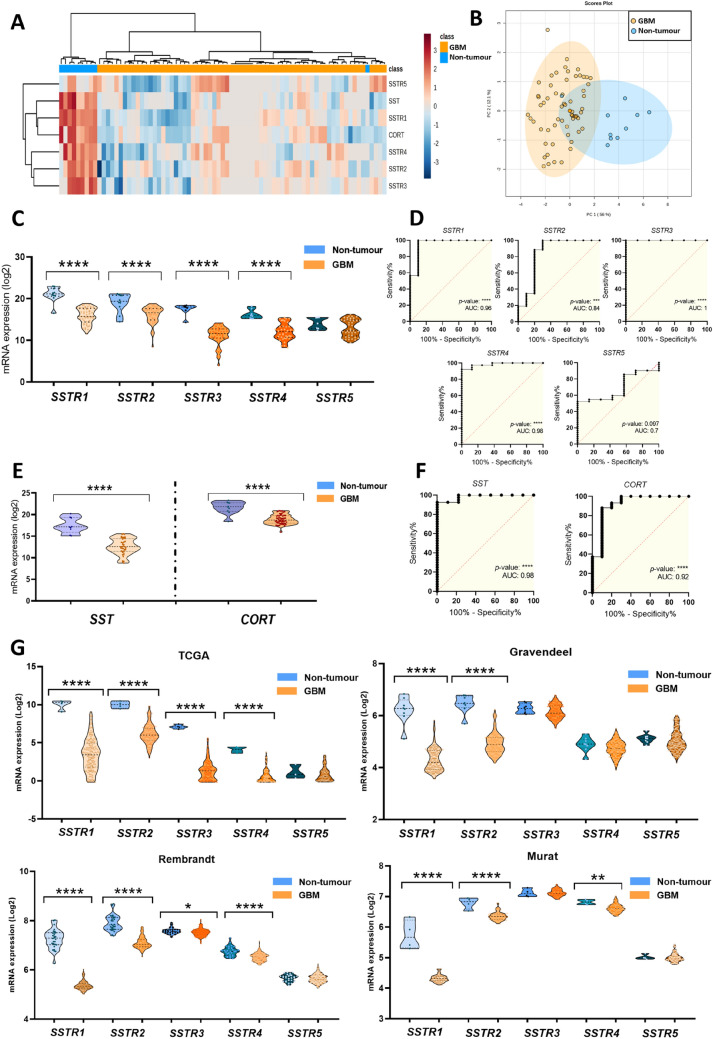
Dysregulation of the SST/CORT-system in GBM patients in our internal cohort. **A** Heatmap generated using the expression levels of all the SSTRs (*SSTR1*-*5*) and ligands (*SST* and *CORT*) in non-tumour samples (*n* = 10) and GBM samples (*n* = 62). **B** Principal components analysis (PCA) of the mRNA expression levels of the SST/CORT-system in the same sample set. **C** mRNA expression levels of *SSTR1-5* comparing non-tumour and GBM samples and **D** their associated ROC curves analysis. **E** mRNA expression levels of the SSTR ligands, *SST* and *CORT*, in non-tumour and GBM samples and **F** their respective ROC curves analysis. **G** mRNA expression levels of *SSTR1-5* comparing non-tumour and GBM samples in TCGA, Rembrandt, Gravendeel, and Murat. Data represent means ± SEM. *P < 0.05, **P < 0.01, ***P < 0.001, ****P < 0.0001 significantly different from control conditions. AUC: Area Under the Curve. See also Supplementary Fig. 1–4

Non-supervised hierarchical analysis based on the expression of all SST/CORT-system components (2 ligands/5 SSTRs) was able to effectively discriminate between GBMs and control samples [internal cohort (Table 1)], except for a single control sample (Fig. 1A). This discrimination was further confirmed by PCA analysis (Fig. 1B). Specifically, we observed an overall downregulation of the SST/CORT-system, with all receptors significantly downregulated except for *SSTR5* (Fig. 1C). Moreover, individual ROC curve analysis of *SSTR1-4* expression was capable of accurately discriminate tumour and non-tumour samples, with an Area Under the Curve (AUC) > 0.8 (Fig. 1D). Likewise, significant low expression of *SST* and *CORT* neuropeptides was also observed in GBM *vs*. control samples (Fig. 1E), with AUC > 0.9, indicating also a high discriminatory capacity of these ligands (Fig. 1F).

These findings were validated in 4 additional human external cohorts (Table 1). Indeed, non-tumour samples clustered closely together in non-supervised heatmaps in these 4 cohorts [TCGA (Fig. S1A; *n* = 156 GBM/*n* = 4 non-tumour samples), Rembrandt (Fig. S2A; *n* = 217 GBM/n = 28 non-tumour samples), Gravendeel (Fig. S3A; *n* = 159 GBM/*n* = 8 non-tumour samples), and Murat (Fig. S4A; *n* = 80 GBM/n = 4 non-tumour samples)]. Likewise, PCA clearly distinguished between GBM and control samples (Fig. S1–S4B, respectively), validating the capacity of the SST/CORT-system components to differentiate between tumour and non-tumour tissues using both Bulk RNA-Seq and microfluidic qPCR technology. Specifically, we found that *SSTR1* and *SSTR2* were the two components consistently downregulated in the 5 independent analysed cohorts (Fig. 1C/-G), while *SSTR3* and *SSTR4* were also frequently downregulated but not in all the cohorts (Fig. 1G). *SSTR5* expression did not change between GBM and non-tumour tissues (Fig. 1C/-1G). Furthermore, similar to our internal cohort (Fig. 1D), *SSTR1* and *SSTR2* AUC analyses were significantly different in all the external cohorts (AUC > 0.95; Fig. S1 –S4C, respectively), remarking the potential of *SSTR1* and *SSTR2* as diagnostic biomarkers of GBM. Finally, a significant downregulation of *SST* and *CORT* was also found in all cohorts (Fig. S1–S4D, respectively), with AUC > 0.9 (Figs. S1–S4E, respectively). Considering that discriminatory capacity might be biased by different confounders clinical and molecular tumour features (Verhaak subtype, *IDH1* mutations, G-CIMP status, MGMT methylation, age, gender), all these different confounders factors were evaluated individually in the TGCA external cohort (*i.e.,* the most clinically/molecularly complete external cohort) and compared with the previous univariate analysis showed above (Supplementary Table S1). These individual clinical/molecular tumour features analyses revealed AUC near to univariate analyses.

### Protein SSTR2 levels are also downregulated in GBM *vs*. control samples

**Fig. 2:**
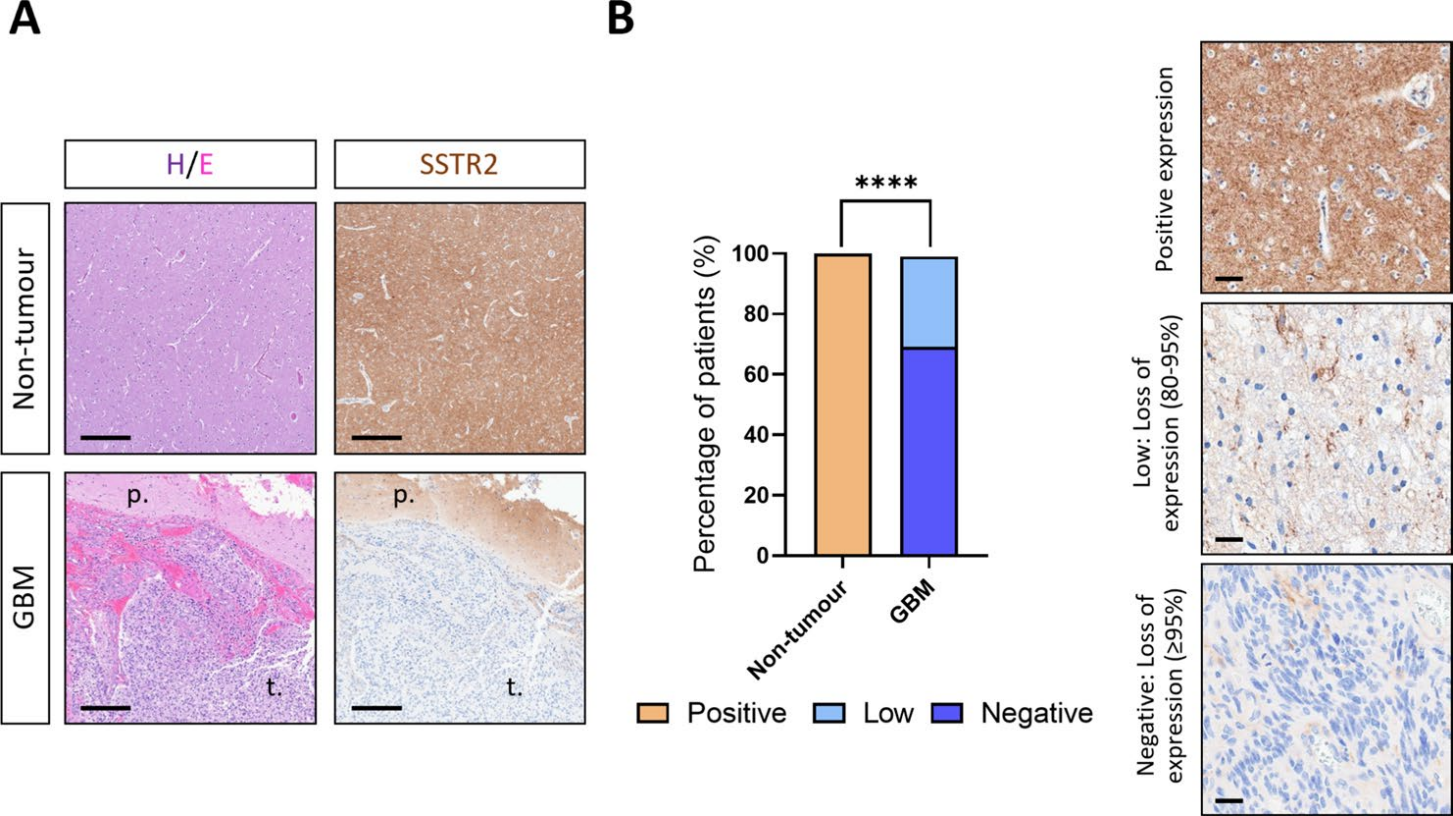
**A** Comparison of SSTR2 protein levels by immunohistochemistry (IHC) between control non-tumour brain tissue and GBM in a representative set of samples (*n* = 4 and *n* = 13, respectively). Representative haematoxylin/eosin and SSTR immunostaining images are shown. Scale bar: 100 μm. t.: tumoural area; p.: peritumoural area. **B** Percentage of patients for each SSTR2 IHC score (left panel), and representative SSTR immunostaining images of different IHC Scores: Positive expression, Low Expression (loss of 80–95% expression), and Negative expression (loss of ≥ 95%) (right panel). Scale bar: 20 μm. The k-square test was used

SSTR1 and SSTR2 IHC analyses were performed in a set of GBM *vs*. control samples using commercially available antibodies previously reported [38]. Unfortunately, reliable immunoreactivity with the SSTR1 antibody (at different concentrations and conditions) could not be achieved in any of the GBM or control tissue samples (including colon and foetal lung tissues used as controls), thereby precluding the possibility of IHC scoring for SSTR1. Representative images of SSTR2 staining are illustrated in Fig. 2A, showing control (upper-panels) and GBM (bottom-panels) samples. Notably, robust SSTR2 expression was observed in all the control tissues (typically localized to the cell membrane or membrane/cytoplasm). In contrast, SSTR2 expression in GBM samples was primarily cytoplasmic, and mainly expressed at low levels with a sparse pattern (Fig. 2B). Among the analysed tumours, nine (69.23%) displayed either no or extremely low cytoplasmic staining, while four tumours (30.77%) showed low cytoplasmic immunoreactivity (< 85% of tumour cells expressing SSTR2). Importantly, none of the tumour tissues exhibited strong immunoreactivity, although intense SSTR2 expression was observed in the peritumoral area (Fig. 2A, bottom-panels).

### Low *SSTR1* and *SSTR2* expression in GBM tissues is associated with worse overall survival and aggressive phenotype in GBM

**Fig. 3:**
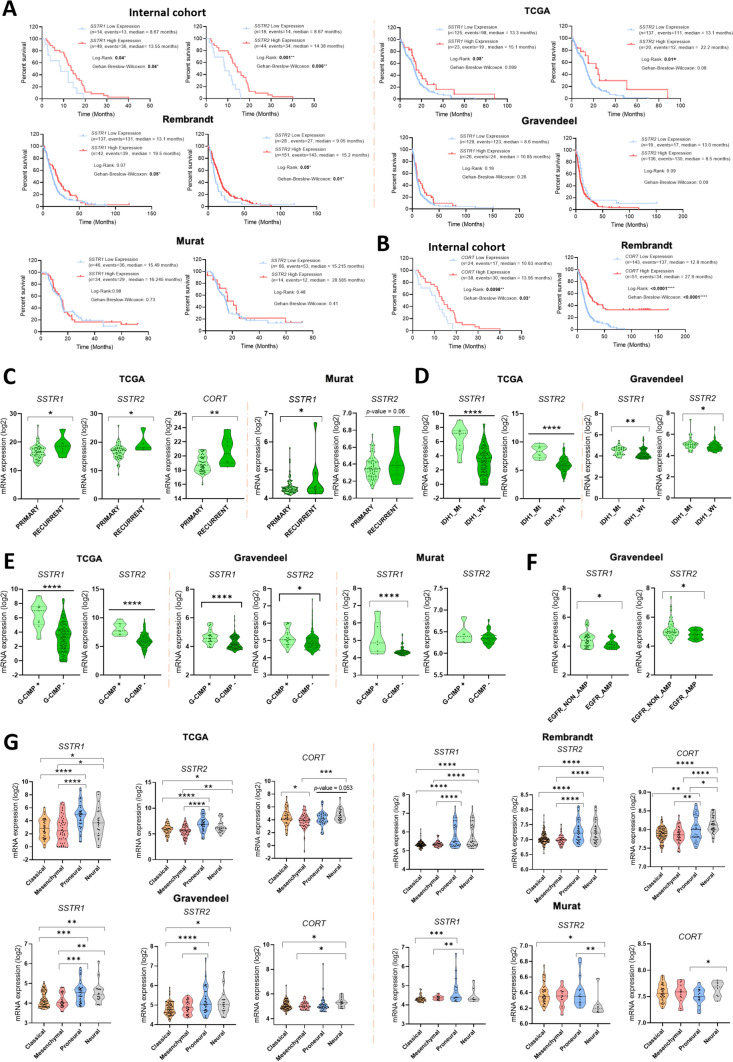
Downregulation of *SSTR1*, *SSTR2,* and *CORT* is associated with key clinical and molecular features related to GBM aggressiveness. **A** Kaplan–Meier survival curves discerning between GBM patients with high and low expression levels of *SSTR1* and *SSTR2* from our internal cohort of patients and from TCGA, Rembrandt, Gravendeel, and Murat datasets. **B** Kaplan–Meier survival curves discerning between GBM patients with high and low expression levels of *CORT* from our internal cohort and Rembrandt dataset. **C** Association between primary or recurrent disease and the expression levels of *SSTR1*, *SSTR2,* and *CORT* from TCGA and *SSTR1* and *SSTR2* in Murat datasets. **D** Comparison of the expression levels of *SSTR1* and *SSTR2* regarding *IDH1* status (Mt, mutant; and Wt, wildtype) from TCGA and Gravendeel datasets. **E** Comparison of the expression levels of *SSTR1* and *SSTR2* concerning the G-CIMP status (+ , positive G-CIMP status, or −, negative G-CIMP status) from TCGA, Rembrandt, Gravendeel, and Murat datasets. **F** Expression levels of *SSTR1* and *SSTR2* in GBM samples divided into carriers of *EGFR* Amplification (AMP) or not (NON_AMP), from Gravendeel dataset. **G** Association between GBM Verhaak molecular subtypes (Classical, Mesenchymal, Proneural, and Neural) and the expression levels of *SSTR1*, *SSTR2*, and *CORT* from TCGA, Rembrandt, Gravendeel, and Murat datasets. *P < 0.05, **P < 0.01, ***P < 0.001, ****P < 0.0001 significantly different from control conditions

Lower *SSTR1* and *SSTR2* mRNA expression levels (SSTR-subtypes consistently downregulated in the 5 analysed independent cohorts) were significantly associated with worse overall-survival (OS) in our internal cohort (Fig. 3A, top-left panel), which was subsequently validated in the TCGA-cohort for *SSTR1* and *SSTR2* (Fig. 3A, top-right panel), and in the Rembrandt-cohort for *SSTR2* [Fig. 3A, bottom-left panel); a trend for significance was also found for *SSTR1* in this cohort and for *SSTR2* in the Gravendeel-cohort (Fig. 3A, bottom-right panel)]. Interestingly, a significant association of lower *CORT* expression, but not *SST* expression (data not shown), with worse OS was observed in our internal cohort, which was also validated in the Rembrandt-cohort (Fig. 3B, left/right-panels).

Based on the previous results, we evaluated the association of *SSTR1*/*SSTR2*/*CORT* with other clinically relevant aggressive parameters (Table 1). A lower *SSTR1* and *SSTR2* expression levels was associated to primary tumours compared to recurrent GBMs in the TCGA/Murat-cohorts (Fig. 3C). Similar association was observed for *CORT* expression in the TGCA-cohort (Fig. 3C). These results were validated in the GLASS dataset in the case of *SSTR1* and *SSTR2* (Fig. S5A). Interestingly, further analyses in the TCGA and Murat datasets showed a worse progression in patients with high expression levels of *SSTR1* and *SSTR2* treated with radiotherapy (RT; Fig. S5B/C, top panels), whereas a better response to the combination of RT + temozolomide (TMZ) was observed in patient with high expression levels of *SSTR1* and *SSTR2* (Fig. S5B/C, bottom panels). Concerning *IDH1*-status (mutations previously related to a better prognosis [42]), low *SSTR1* and *SSTR2* expression was robustly associated with *IDH1*-wildtype patients in the TCGA/Gravendeel-cohorts (Fig. 3D). Additionally, G-CIMP negative tumours (representing a more aggressive GBM phenotype) have significantly lower expression levels of *SSTR1* and/or *SSTR2* (Fig. 3E), but not *CORT* (data not shown), in the TCGA/Gravendeel/Murat-cohorts. Moreover, we observed that lower *SSTR1* and *SSTR2*, but not *CORT*, expression levels were also displayed in tumours harbouring *EGFR* amplification, another aggressive sign in GBM [43, 44], in the Gravendeel-cohort (Fig. 3F). Furthermore, when evaluating Verhaak-subtypes (widely reported to be associated with OS [45]), low expression levels of *SSTR1*, *SSTR2,* and/or *CORT* were also significantly associated with classical and mesenchymal subtypes (which present the worst OS) *vs*. proneural and/or neural subtypes in the TCGA/Rembrandt/Gravendeel, and/or Murat-cohorts (Fig. 3G). Finally, no association was observed between *SSTR1*/*SSTR2*/*CORT* expression and the methylation status of *MGMT*-promoter (data not shown).

### Distribution of SSTRs in different GBM populations at single cell level, and antitumor actions of somatostatin analogs and selective SSTRs agonists on patient-derived primary GBM cell-cultures

**Fig. 4:**
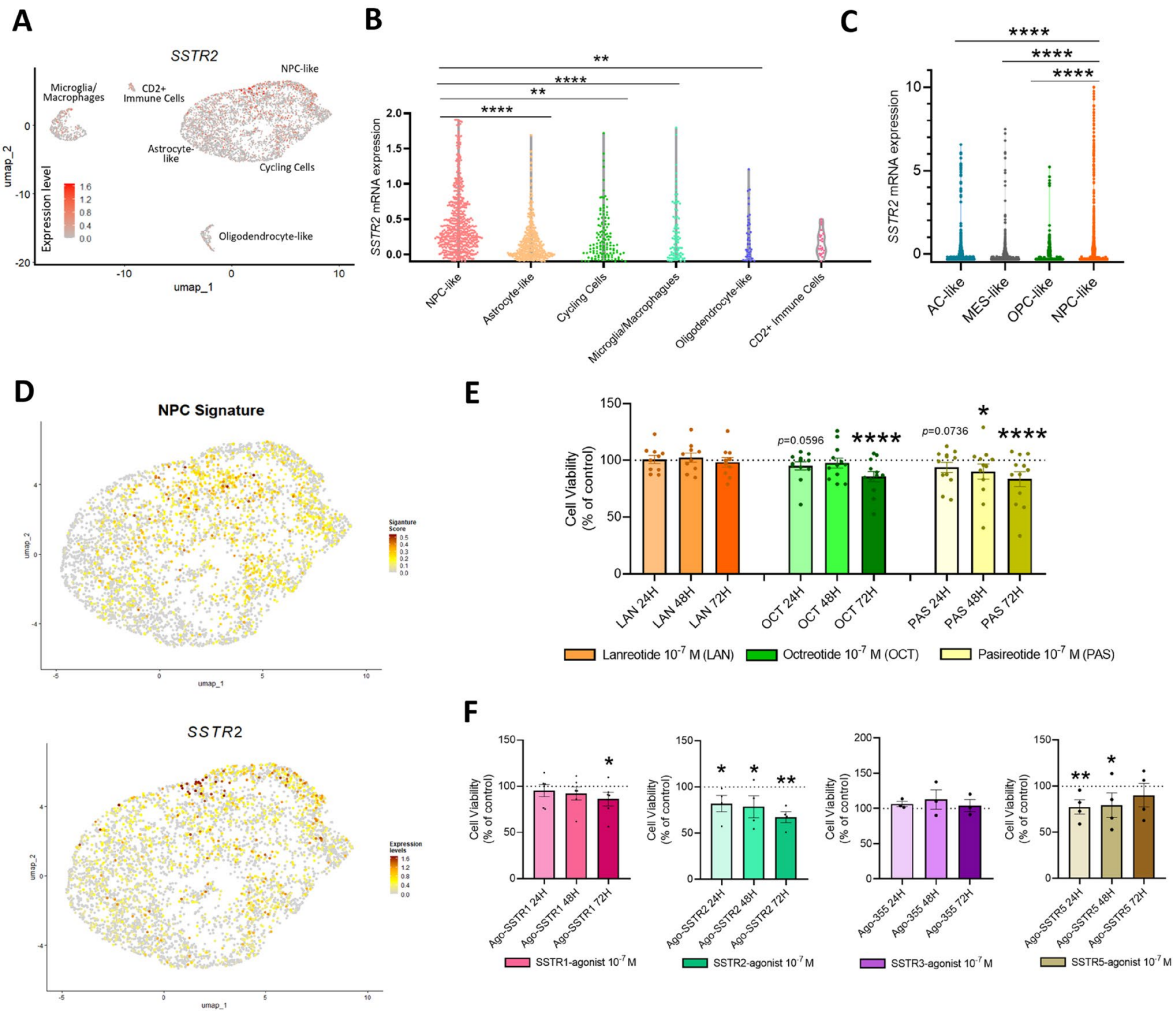
Distribution of SSTR2 in different GBM populations at single cell level, and effects of treatment with SSAs and SSTR agonists on primary patient-derived GBM cell cultures. **A** Distribution of SSTR2 expression in distinctive Uniform Manifold Approximation and Projection (UMAP) cluster at single cell level. **B** SSTR2 expression across different intra-tumour cell subtypes identified. **C** SSTR2 expression across different GBM cell transcriptional programs. **D** Gene expression of NPC signature (based on the mRNA expression levels of *SOX2*, *NES*, and *ASCL1* markers; top) and *SSTR2* (bottom) in tumour cells. Proliferation/viability rates in primary patient-derived GBM cell cultures treated with (**E**) SSAs: Lanreotide (LAN, *n* = 10), Octreotide (OCT,* n* = 12), and Pasireotide (PAS,* n* = 12); and **F** SSTR1-agonist (Ago-SSTR1,* n* = 5), SSTR2-agonist (Ago-SSTR2,* n* = 4), SSTR3-agonist (Ago-355,* n* = 3), and SSTR5-agonists (Ago-SSTR5,* n* = 4). Each dot represents a single patient in **E** and **F**. Data represent means ± SEM. *P < 0.05; **P < 0.01; ****P < 0.0001, significantly different from control conditions

As previously observed in whole tissues, we found that SST/CORT-system (mainly *SSTR2*) was scarcely expressed in all the GBM microenvironment. Concretely, *SSTR2* showed a low expression in a limited number of cells (Fig. 4A). Interestingly, we observed that the population expressing the higher *SSTR2* levels was the NPC population (Fig. 4B). Similarly, the transcriptomic state with the highest *SSTR2* levels was also NPC-like (compared to MES, OPC, AC-like; Fig. 4C). Remarkably, when selecting only the tumour cells in the dataset, we also observed a similar expression pattern in NPC signature (based on *SOX2*, *NES*, and *ASCL1* genes) and *SSTR2* (Fig. 4D), supporting the putative relationship between this NPC population and SSTR2 levels.

Our results demonstrated that a dose of 10^−7^ M of octreotide and pasireotide, but not of lanreotide, significantly reduced the proliferation rate of primary GBM cell-cultures, with pasireotide showing earlier time-effects (at 48/72 h, and a trend for significance at 24 h) compared to octreotide (showing only significant reduction at 72 h) (Fig. 4E). Interestingly, octreotide and/or pasireotide showed a higher antiproliferative effect in IDHwt patients compared to IDHmt (both drugs; Fig. S5D), and in recurrent than primary tumours (only octreotide reached statistical significance; Fig. S5E). Moreover, we evaluated apoptotic activity in primary GBM cell cultures in response to octreotide and pasireotide (48 h of incubation) showing a slight, but not significant, increase in apoptotic activity in response to pasireotide (Fig. S5F), which is in accordance with the slight (not significant) change observed at the mRNA levels of some apoptotic markers in response to these treatments (Fig. S5G). Additionally, treatment with specific agonists for SSTR1, SSTR2, and SSTR5, but not for SSTR3, significantly decreased the proliferation rate of GBM cells at different times (24–48-72 h) depending on the agonist (Fig. 4F), being the most effective SSTR2-agonist (32.6% reduction at 72 h), followed by SSTR5-agonist (22.4% reduction at 24 h), and SSTR1-agonist (13.9% reduction at 72 h) (Fig. 4F).

### Octreotide and pasireotide treatment modulated the expression and phosphorylation levels of different components associated with key oncogenic pathways in GBM cells

**Fig. 5:**
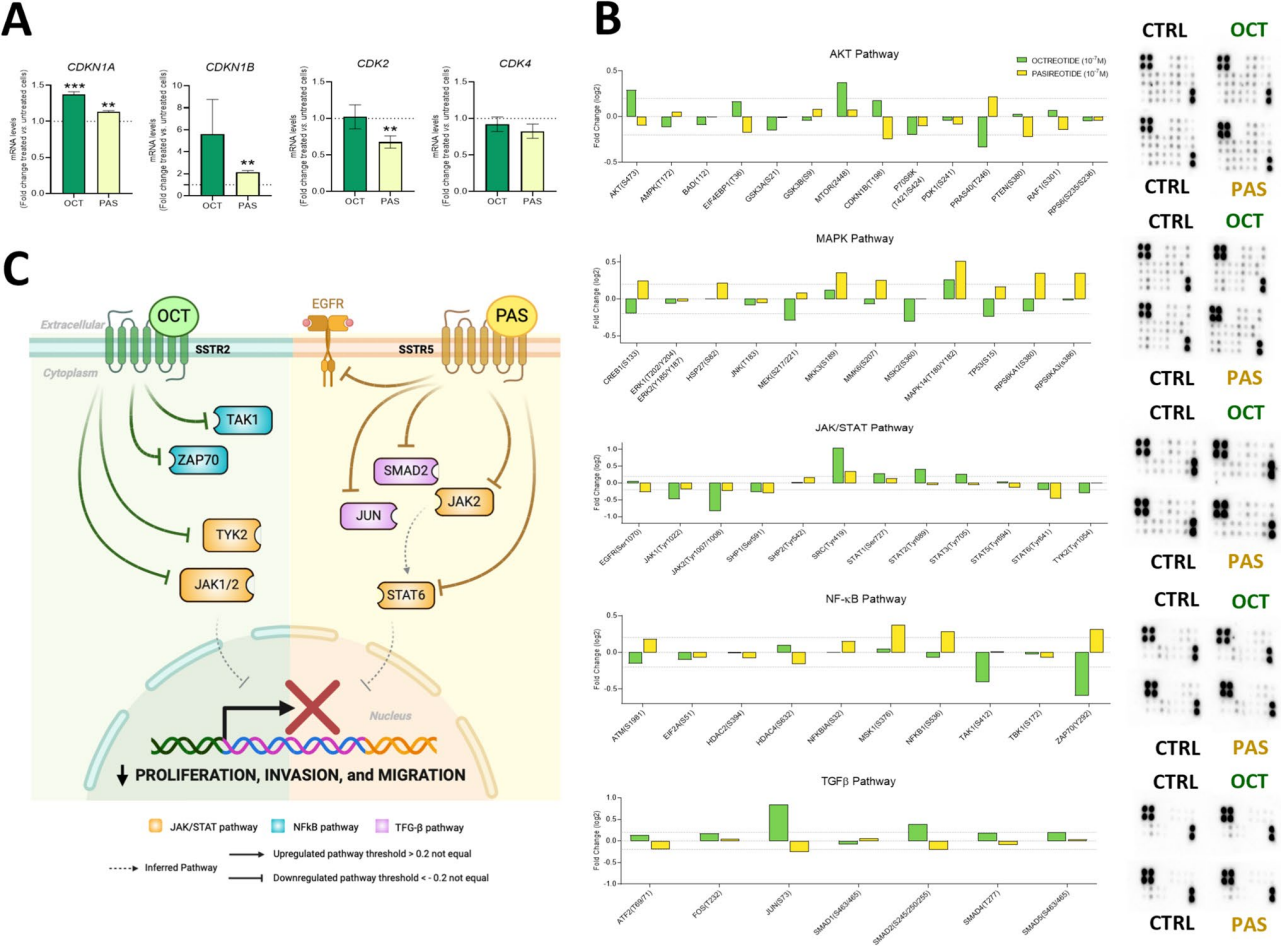
Molecular alterations after treatment with octreotide and pasireotide on primary patient-derived GBM cell cultures. **A** Fold change of mRNA copy number (measured by qPCR) of cell-cycle cyclin kinase inhibitors (*CDKN1A* and *CDKN1B*) and cell-cycle activators (*CDK2* and *CDK4*) in primary GBM cell culture samples treated with octreotide (OCT) and pasireotide (PAS) compared to control-treated samples (*n* = 5) Data represents means ± SEM. **B** Individual phosphorylation protein levels after OCT-treated and PAS-treated *vs*. control condition in primary GBM cell cultures [threshold: log2(FC) = 0.2] (Left panel) and the membranes showing the spots quantified to study the phosphorylation level of 55 proteins under different experimental conditions [OCT-treated, PAS-treated *vs*. control-treated primary GBM cell cultures (Right panel)]. Data represents means. *P < 0.05; **P < 0.01; ***P < 0.001; ****P < 0.0001, significantly different from control conditions. See also Supplementary Fig. S6. **C** Schematic representation of altered signalling pathways after octreotide (green) and pasireotide (yellow) treatments in primary GBM cell cultures; generated by BioRender.com

Due to limitations in number of cells obtained after the tissue dispersion of each patient´s sample, we only were able to select/test octreotide and pasireotide effects (currently used in clinical practice) on molecular/signalling endpoints. Octreotide-/pasireotide-treated cells showed higher mRNA levels of two well-known cell-cycle brakes, *CDKN1A* (significant in both cases) and *CDKN1B* (significant only in pasireotide-treated cells), whereas *CDK2* was downregulated in pasireotide cells (Fig. 5A). Additionally, in order to interrogate potential signalling mechanisms triggered by octreotide and pasireotide, we examined the phosphorylation levels of multiple proteins involved in 5 critical oncogenic pathways (AKT/MAPK/JAK-STAT/NF-κB/TGF-β; for details, see Fig. S6A). Specifically, octreotide antitumour effects might be driven mainly through the decrease of JAK/STAT phosphorylation levels (by modulating JAK1/JAK2/TYK2) and NFκB-pathway (by modulating TAK1/ZAP70) (Fig. 5B/5C). Conversely, pasireotide antitumour effects might be driven mainly through the modulation of JAK/STAT phosphorylation levels (by affecting EGFR/JAK2/STAT6) and TGFβ-pathway (by modulating JUN/SMAD2) (Fig. 5B/5C). Interestingly, both pathways were overrepresented in mesenchymal and classical Verhaak subtypes compared to proneural and neural GBMs (Fig. S6B). Moreover, in line with the downregulation observed in the phosphorylation levels after octreotide and pasireotide treatments, we found a negative correlation between *SSTR2* or *SSTR5* expression levels and both JAK/STAT and NF-κB scores (Fig. S6C).

## Discussion

There is a critical need to identify efficient molecular targets for GBM patients, to improve diagnosis, to predict their tumour behaviour and prognosis, but specially to provide useful tools to develop novel therapeutic approaches. An emerging approach against several cancer types is based on repositioning drugs already approved for other pathologies since they are faster and easier to translate to clinical practice than new drugs. In fact, SST/CORT-system has been widely reported as a source of diagnostic/prognostic biomarkers, and therapeutic targets across a wide range of cancers [16, 24, 46]. However, studies in brain tumours using limited human cohorts have rendered controversial results [25–27, 47], and the in vitro approaches have been exclusively performed using only cell-line models [28, 48, 49]. To the best of our knowledge, this is the first report wherein the expression of the SST/CORT-system (ligands/SSTR1-5) has been thoroughly and quantitatively (normalized mRNA copy-number) analysed in a relatively large series of GBM samples. Specifically, we observed a differential expression pattern in GBM-tissues (*SSTR2 *> *SSTR1* > *SSTR5* > *SSTR4* > *SSTR3*) compared to the control-tissues (*SSTR1* > *SSTR2* > *SSTR3* > *SSTR4* > *SSTR5*). Moreover, we demonstrated the existence of an overall decrease in the expression of all SST/CORT-system components [ligands (SST/CORT) and SSTRs] in GBM-samples, compared to control-samples, being this downregulation statistically significant for all components, except for *SSTR5*. This might be clinically relevant, as SSAs responsiveness is critically dependent on the presence of SSTRs, and because SSAs treatment (which preferential bind to SSTR2) has become the mainstay of medical therapy for tumour control in different neuroendocrine-pathologies [18, 50]. Our observations favourably compare with available data obtained from 4 additional cohorts of GBM-tissues (TCGA [33]/Rembrandt [34]/Gravendeel [35]/Murat [36]), especially for *SSTR1* and *SSTR2*, and with previous reports involving some of these components in intracranial tumours [25, 51, 52]. In fact, individual expression levels of these components revealed that *SSTR1*, *SSTR2*, *SST*, and *CORT* have discriminatory capacity comparing GBM and control samples, suggesting a potential value as diagnostic-biomarkers. However, it should be noted that diagnostic markers that are downregulated in tumours pose challenges in terms of sensitivity and, more notably, in establishing a precise cut-off to distinguish between tumour and non-tumour tissue. Therefore, although our findings indicate that nearly all tumour samples effectively differentiate GBM from non-tumour controls, caution should be exercised with the translational utility of these components of the SST/CORT-system as diagnostic tools, an aspect that would need further exploration.

Another relevant finding of our study was that *SSTR1* and *SSTR2* expression in GBM-tissues (dominant SSTR-subtypes expressed in GBM-samples and consistently downregulated across the 5 human analysed cohorts), were associated with malignant features (*i.e.,* OS/recurrence/*IDH1*-status/GCIMP-status/molecular Verhaak-subtypes/*EGFR*-mutations). Specifically, *SSTR1/SSTR2*-expression is directly associated with a worse OS-rate, certainly, the main clinical problem in GBM-patients. Additionally, *SSTR1* and/or *SSTR2* expression was also associated with different aggressiveness parameters (*i.e., IDH1*-wildtype [53]/GCIMP-negative [54]/classical-mesenchymal subtypes [55]/*EGFR*-amplified samples [43, 44]). Conversely, recurrent-tumours (more aggressive than primary-tumours [56, 57]) showed higher *SSTR1* and/or *SSTR2* levels. Herein, it is tempting to speculate that local therapies after surgery (*i.e.,* radiotherapy and/or chemotherapy) might indirectly alter the receptor expression levels, as it has been previously reported a SSTRs-upregulation (mainly SSTR2) after treatment with temozolomide [58]. However, further studies would be necessary to unequivocally corroborate this idea. Nonetheless, our data show that the main clinical correlations were associated with *SSTR1* and *SSTR2* expression in GBM, suggesting also a potential prognostic-value of these receptors in GBM, which might be a common cellular/molecular-signature across various tumour-types that also have proposed a potential prognostic value of these receptors (*e.g.,* colon cancer [59] and prostate cancer [60] for SSTR1, and rectal neuroendocrine-tumours [61], meningioma [62, 63], and nasopharyngeal carcinoma [64] for SSTR2).

Supporting this latter idea is our observation based on single-cell data demonstrating that *SSTR2* was expressed across all GBM cell-populations/states, being higher in cells expressing a proliferative neural progenitors-like transcriptional program which have been reported as relevant in terms of tumourigenesis/progression/therapy-resistance [65]. Moreover, a similar distribution pattern was found for *SSTR2* and the expression of key NPC markers in tumour cells. This might be pathophysiological relevant since NPCs have been reported to be relevant in terms of tumourigenesis/progression/therapy-resistance [65]. Concretely, plasticity of neural stem cells might develop glioma stem cells, depending on key mutations in protooncogenes or tumour-suppressor genes, due to their relevance in differentiation and migration processes in both physiological and pathological conditions [65, 66]. Therefore, these data might have a potential translational/oncogenic implication since the current therapeutic strategies for GBM are not efficient at reducing tumour volume/growth or augmenting survival-rate, which is likely due, in part, to the resistance acquired by tumours, particularly by neural progenitors-cells, to the available therapies [65]. Indeed, a potential relevance in therapeutic response is also supported by bulk results showing significant differences in the expression levels of *SSTR1* and *SSTR2* in primary *vs*. recurrent samples and in response to RT/TMZ. Based on these data, and knowing that even cells with low SSTRs expression can exhibit a significant SSAs-response [67], we next explored the direct effects of different SSAs (first/second-generation), and selective SSTR1/SSTR2/SSTR5-agonists on the proliferation-rate of patient-derived primary GBM cell-cultures. Based on current evidence, our results are the first to demonstrate that human GBM-cells are quite responsive in reducing the proliferation-rate of GBM-cells to first-/second-generation of SSAs [octreotide (mainly targeting SSTR2, with moderate affinity for SSTR5 and weak interaction with SSTR3 [68]) and pasireotide (a multi-SSTR agonist with high binding affinity for SSTR1/2/3/5 [68])], but not to SST and CORT (data not shown) or lanreotide (mainly targeting SSTR2 but with a slightly more pronounced affinity to SSTR5 compared with octreotide [68]). Therefore, these data suggest that SSAs exert antiproliferative effects on patient-derived primary GBM-cells, with different effects depending on *IDH1* status and recurrence, opening new avenues to explore their potential as novel targeting therapy for patients with GBM from a personalized perspective. In fact, we had the opportunity to use specific SSTRs-agonists and found that the agonists targeting SSTR1, SSTR2, and SSTR5, but not SSTR3, also exerted antiproliferative effects in GBM-cells, being the most effective the SSTR2-agonist. Overall, these data might provide a scientific rationale for a randomized controlled trial of SSAs (especially those that preferentially bind to SSTR1 and SSTR2) as an adjuvant treatment in GBM. In line with this, differences among SSAs and SSTR-agonists effects have been previously reported in other tumour and non-tumour contexts in terms of functional relevance and signalling pathways [41, 69, 70], which might be due in part to the multi-receptor binding capacity of pasireotide *vs.* octreotide but the involvement of other major and complex molecular events should be not discarded [41, 71–73].

Then, we next interrogated the mechanisms underlying the antiproliferative response to octreotide and pasireotide by exploring an ample range of signalling-pathways. Octreotide and pasireotide treatment increased *CDKN1A/B* (cell cycle-brakes [74]) mRNA levels, which is consistent with reports in other tumour types reporting antiproliferative effects through the reduction of their respective proteins (p21 and p27) [75–78]. Interestingly, in pasireotide-treated cells, a significant decrease in CDKN1B phosphorylation levels at residue T198 (responsible for mislocalizing this protein at cytosol and avoiding its arresting effects [79]) was observed, suggesting that, in accordance with the increase in *CDKN1B* mRNA, p27 could be a potential target of the antiproliferative effect induced by pasireotide. Moreover, we observed a decrease in JAK/STAT phosphorylation levels of membrane receptors (*i.e.,* JAK1/JAK2/TYK1) in response to octreotide-treatment compared to control cells. In line with this observation, this signalling-pathway has been previously reported to be relevant to promote cell-proliferation/invasion/progression in GBM [80–82]. Specifically, activation of JAK receptors was associated with classical GBM-subtype [80, 81], which could be in concordance with the observed association between this aggressive subtype and the downregulation of SSTR2 expression (main octreotide target). Similarly, octreotide-treatment also reduced phosphorylation levels of key intermediates in the NF-κB pathway (*i.e.,* ZAP70/TAK1), whose activation has been linked to GBM-growth/invasion [83–85]. Furthermore, this pathway was also associated with mesenchymal GBM-subtype [83], which might be related to the previously observed association between SSTR2 downregulation and this GBM-subtype. Interestingly, JAK/STAT and NF-κB activation in GBM have been related to resistance to chemo- [81] and radio-therapy [86], respectively. Additionally, the negative correlation observed between the expression levels of *SSTR2* and these two signalling pathways supports the idea that *SSTR2* activation might be involved in the blockade of these relevant routes. Therefore, when view together these data invite us to hypothesize a potential synergistic effect by combining this SSA with the standard of care in these tumours. Conversely, pasireotide-treatment activated MAPK [signalling-pathway associated with the physiological SSTR5-activation (main pasireotide target)] [87]. Moreover, antitumour pasireotide-effects might be also explained by the observed decrease in key phosphoproteins involved in JAK/STAT-pathway, highlighting EGFR (important pathophysiological receptor in GBM [44]), the receptor JAK2, and other downstream intermediates, such as STAT6, whose relevance in GBM has been reported, promoting GBM cell-proliferation/migration/invasion [80]. Interestingly, we also observed that *SSTR2* and *SSTR5* expression levels are negatively correlated to JAK/STAT score, in accordance with the reduction in the phosphorylation levels of different JAK/STAT elements. Finally, it is also noteworthy that pasireotide-treatment decreased the phosphorylation levels of key factors involved in the TGF-β signalling-pathway (associated with aggressiveness in gliomas [88, 89]), such as JUN (responsible for tumour-invasion [89]). Considering that current therapies and clinical trials are focussed on targeting specific and relevant pathways in GBM physiopathology, including the use of TGF-β inhibitors in gliomas (*e.g.,* NCT01582269, NCT01682187) [90, 91], we speculate that combining TGF-β inhibitors and SSAs might be a potential therapeutic approach worth to be tested in specific subsets of GBM patients. Overall, these results open a new research avenue in the study of GBM suggesting that specific SSAs with high SSTR1/2/5 binding-affinity might serve as a therapeutic option in GBM-patients.

## Conclusions

In summary, this study reveals a drastic downregulation of the whole SST/CORT-system in GBM compared to non-tumour brain samples. Although the SST/CORT-system role in clinical practice needs to be further explored with controlled clinical-trials, our results open a new research avenue in the GBM-field by demonstrating: (1) a consistent SSTR1/SSTR2 expression downregulation in all the analysed human-cohorts with a strong clinical association with the most aggressive GBM phenotype, and with overall-survival/recurrence/molecular-subtypes, positioning both SSTR-subtypes as new potential prognostic biomarker; and (2) that the use of SSAs exhibit clear antitumour effects in primary patient-derived GBM cells through the modulation of key signalling-pathways associated to GBM development/progression/aggressiveness, opening new source of molecular targets that can be targeted alone or in combination with the standard of care to combat GBMs.

## Supplementary Information

Below is the link to the electronic supplementary material.

Supplementary file1 (DOCX 11260 KB)

## Data Availability

External bulk-RNAseq data analyzed in the present study are available in GlioVis-Tools (http://gliovis.bioinfo.cnio.es), and GLASS Consortium (https://glass-consortium.org/); and single-cell RNAseq data in the single-cell Portal-Broad Institute (https://singlecell.broadinstitute.org). The datasets generated and/or analyzed during the current study are available from the corresponding author upon reasonable request.

## Abbreviations

CORT: Cortistatin
GBM: Glioblastoma multiforme
IDH: Isocitrate dehydrogenase
MGMT: Methyl-guanine methyltransferase
NPC: Neural progenitor cell
OS: Overall survival
ROC: Receiver operating characteristic
SSA: Somatostatin analog
SST: Somatostatin
SSTR: Somatostatin receptor
