# Supplementary material for: Assessing the diagnostic, prognostic, and therapeutic potential of the somatostatin/cortistatin system in glioblastoma
Source: Cell Mol Life Sci. 2025 Apr 23;82(1):173. doi: 10.1007/s00018-025-05687-9 (PMC12018673; doi:10.1007/s00018-025-05687-9)
Supplement: Supplementary file 1 — Supplementary file1 (DOCX 11260 KB) [file 18_2025_5687_MOESM1_ESM.docx]

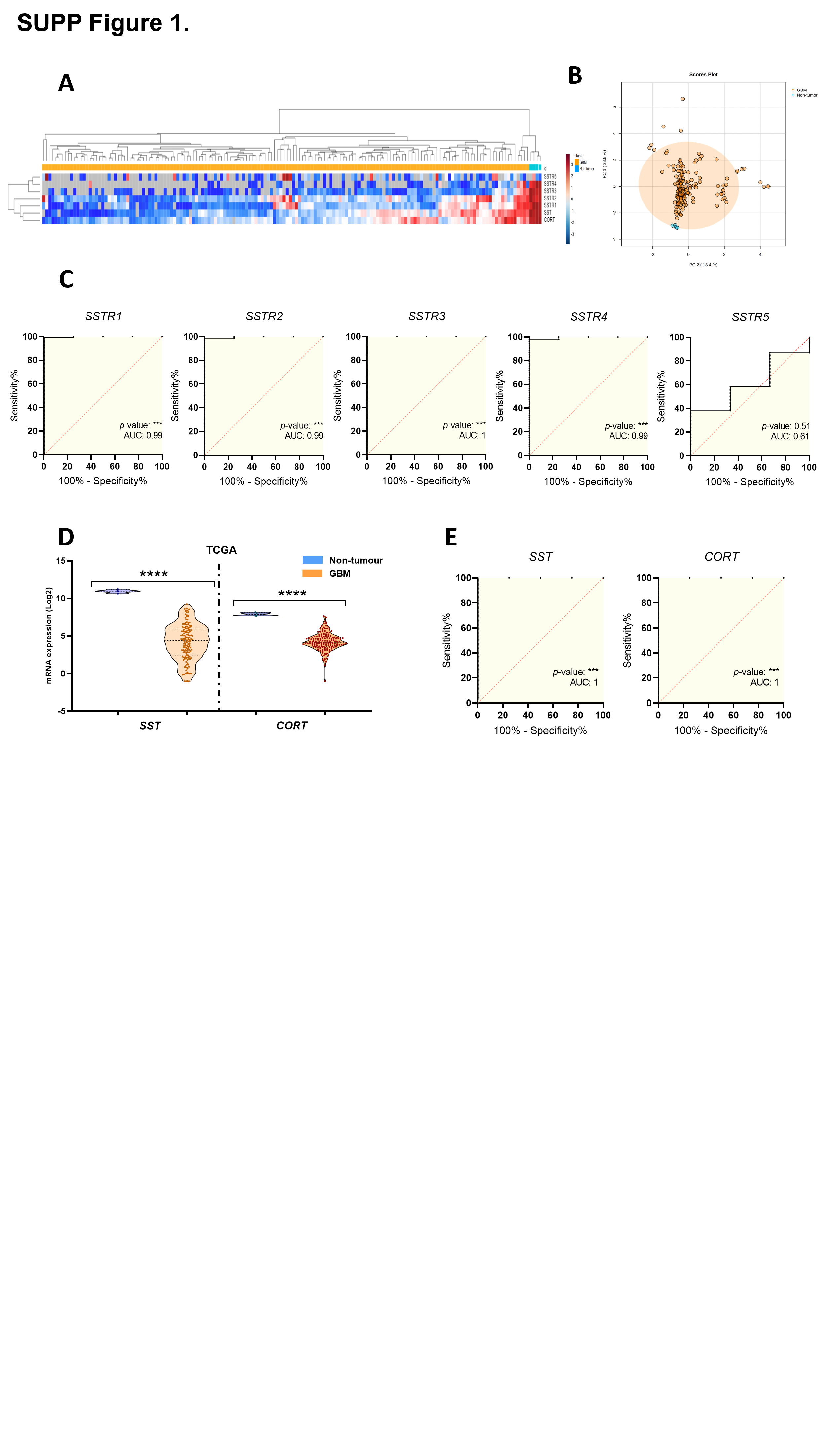
**Fig. S1. Dysregulation of the SST/CORT system in GBM patients is validated in TCGA external human cohort.** (**A**) Heatmap generated using the expression levels of all the SSTRs (*SSTR1*-*5*) and ligands (*SST* and *CORT*) in non-tumour samples (*n* = 4) and Glioblastomas (GBMs) samples (*n* = 156). (**B**) Principal components analysis (PCA) of the mRNA expression levels of the SST/CORT system in the same sample set. (**C**) ROC curves analysis from *SSTR1-5* mRNA expression levels comparing non-tumour and GBM samples. (**D**) mRNA expression levels of the SSTR ligands, *SST* and *CORT*, in non-tumour and GBM samples and (**E**) their respective ROC curves analysis. Data represent means ± SEM. ***P < 0.001, ****P < 0.0001 significantly different from control conditions. AUC: Area Under the Curve.


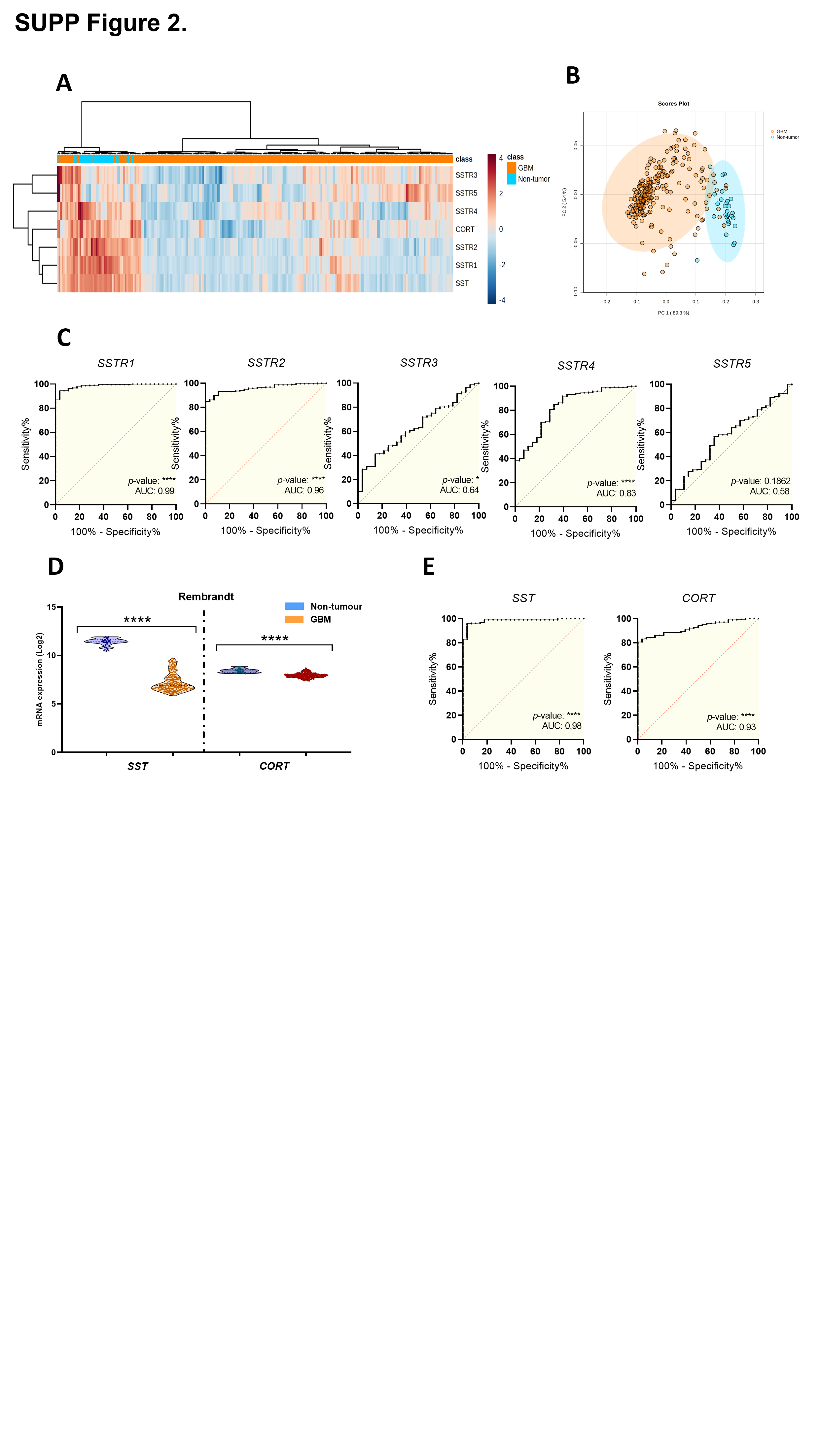
**Fig. S2. Dysregulation of the SST/CORT system in GBM patients is validated in Rembrandt external human cohort.** (**A**) Heatmap generated using the expression levels of all the SSTRs (*SSTR1*-*5*) and ligands *SST* and *CORT*) in non-tumour samples (*n* = 28) and Glioblastomas (GBMs) samples (*n* = 217). (**B**) Principal components analysis (PCA) of the mRNA expression levels of the SST/CORT system in the same sample set. (**C**) ROC curves analysis from *SSTR1-5* mRNA expression levels comparing non-tumour and GBM samples. (**D**) mRNA expression levels of the SSTR ligands, *SST* and *CORT*, in non-tumour and GBM samples and (**E**) their respective ROC curves analysis. Data represent means ± SEM. *P < 0.05, ****P < 0.0001 significantly different from control conditions. AUC: Area Under the Curve.


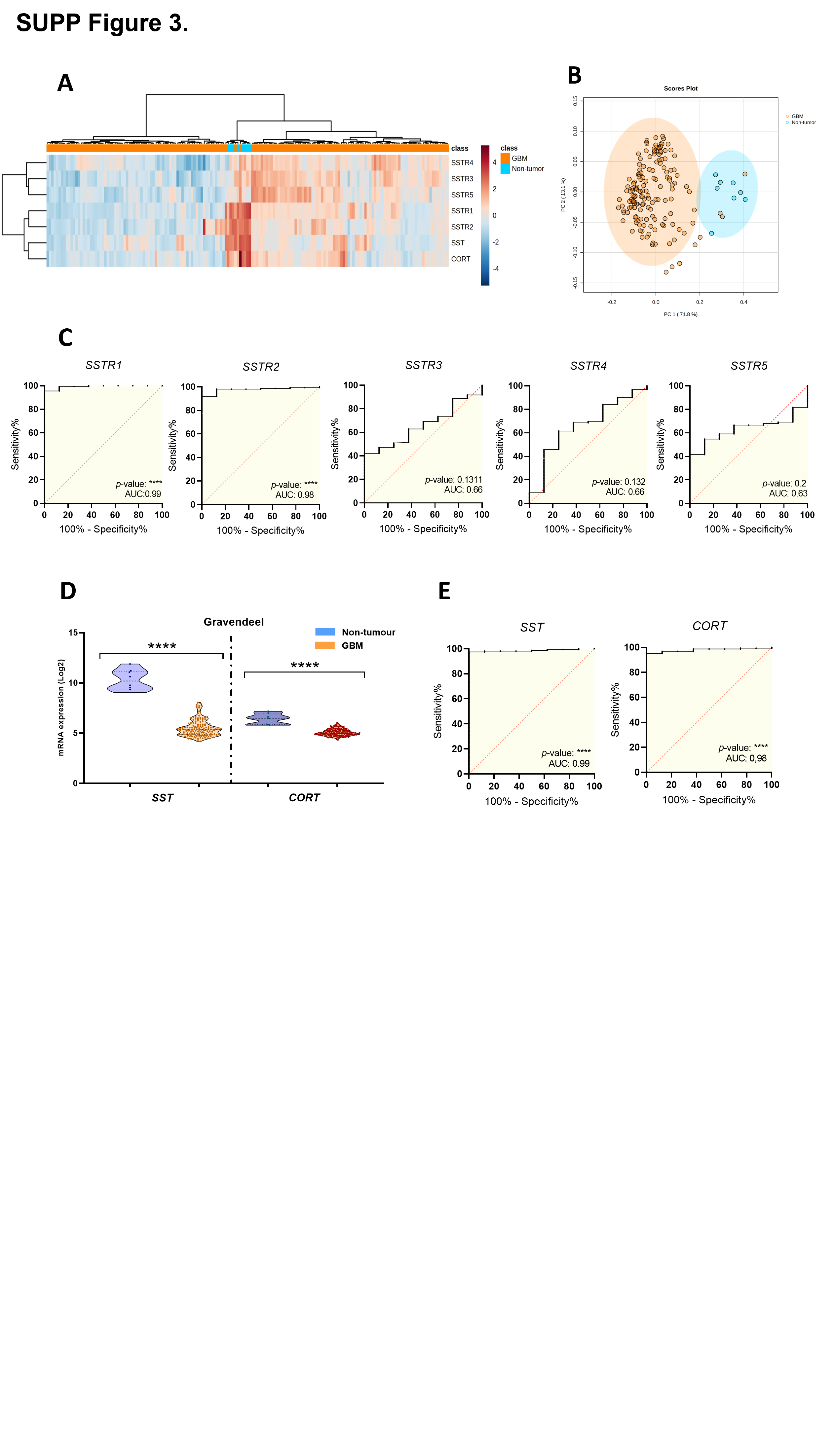
**Fig. S3. Dysregulation of the SST/CORT system in GBM patients is validated in Gravendeel external human cohort.** (**A**) Heatmap generated using the expression levels of all the SSTRs (*SSTR1*-*5*) and ligands (*SST* and *CORT*) in non-tumour samples (*n* = 8) and Glioblastomas (GBMs) samples (*n* = 159). (**B**) Principal components analysis (PCA) of the mRNA expression levels of the SST/CORT system in the same sample set. (**C**) ROC curves analysis from *SSTR1-5* mRNA expression levels comparing non-tumour and GBM samples. (**D**) mRNA expression levels of the SSTR ligands, *SST* and *CORT*, in non-tumour and GBM samples and (E) their respective ROC curves analysis. Data represent means ± SEM. ****P < 0.0001 significantly different from control conditions. AUC: Area Under the Curve.


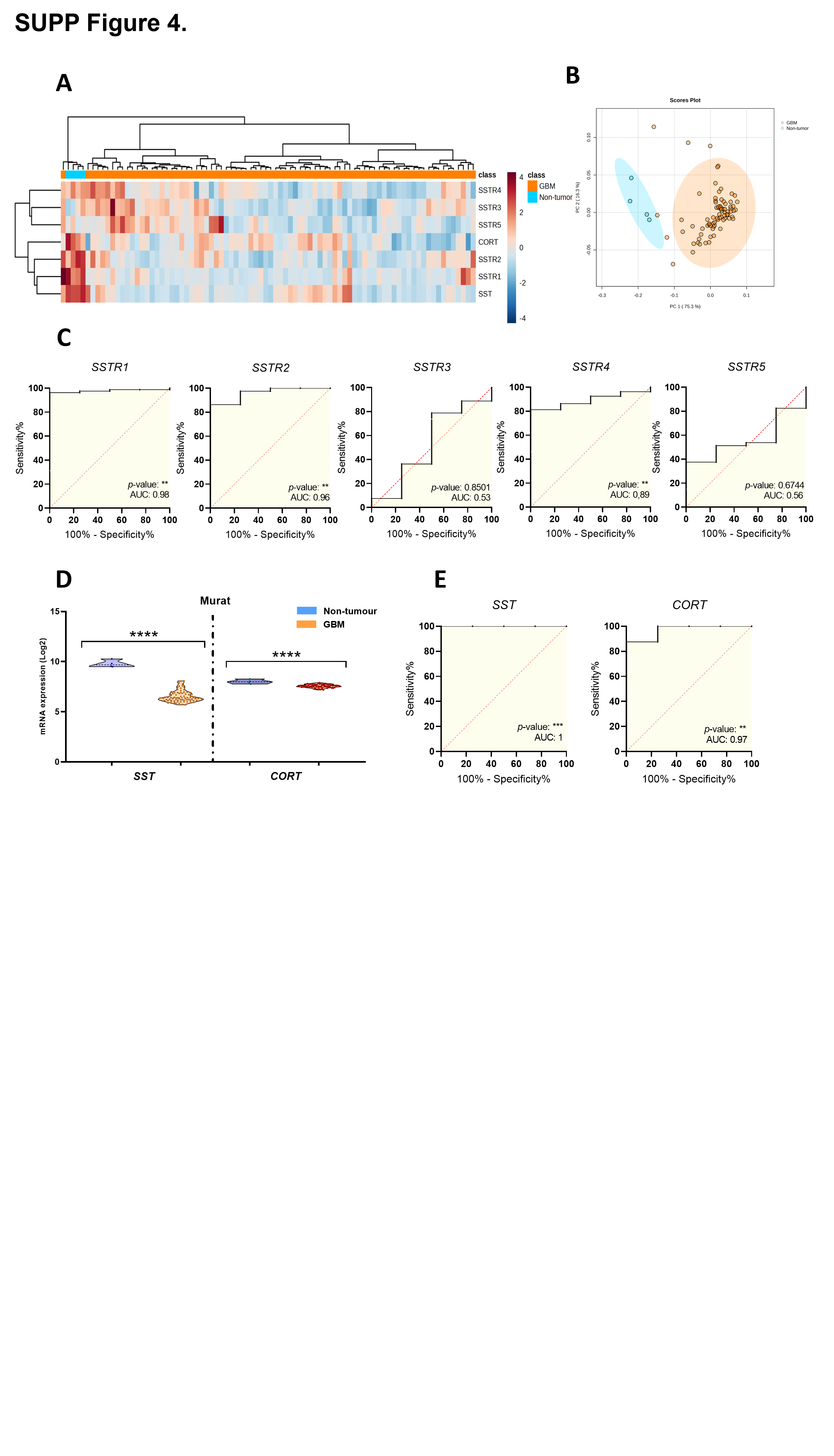
**Fig. S4. Dysregulation of the SST/CORT system in GBM patients is validated in Murat external human cohort.** (**A**) Heatmap generated using the expression levels of all the SSTRs (*SSTR1*-*5*) and ligands (*SST* and *CORT*) in non-tumour samples (*n* = 4) and Glioblastomas (GBMs) samples (*n* = 80). (**B**) Principal components analysis (PCA) of the mRNA expression levels of the SST/CORT system in the same sample set. (**C**) ROC curves analysis from *SSTR1-5* mRNA expression levels comparing non-tumour and GBM samples. (**D**) mRNA expression levels of the SSTR ligands, *SST* and *CORT*, in non-tumour and GBM samples and (**E**) their respective ROC curves analysis. Data represent means ± SEM. *P < 0.05, ***P < 0.001, ****P < 0.0001 significantly different from control conditions. AUC: Area Under the Curve.


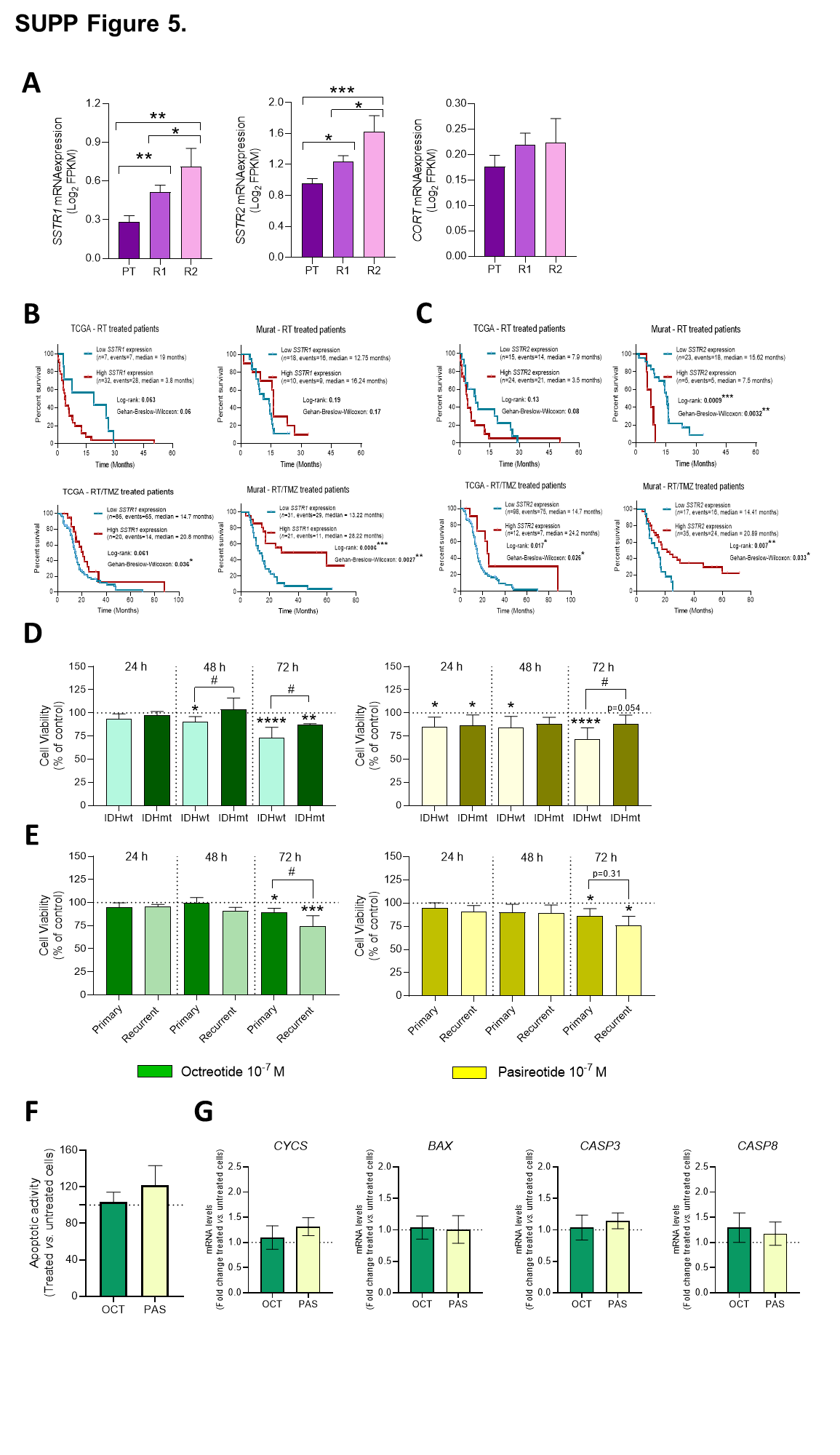


**Fig. S5.** (**A**) Expression levels of each component of *SSTR1*, *SSTR2* and *CORT* in primary tumor samples (PT), and in patients with first recurrence (R1) and second recurrence (R2). Data obtained from the GLASS dataset. Kaplan-Meier survival curves discerning between GBM patients with high and low expression levels of *SSTR1* (**B**) or *SSTR2* (**C**) and response to different therapeutic strategies [only Radiotherapy (RT, top-panels) or combination of RT+Temozolomide (RT/TMZ, bottom-panels) using two available human external cohorts [TCGA (left) and Murat (right)]. Proliferation/viability rates in primary patient-derived GBM cell cultures treated with octreotide (green; left) and pasireotide (yellow; right) considering IDH1 status (**D**) and recurrence (**E**). (**F**) Apoptotic activity of primary GBM cell cultures after 48 h of incubation with octreotide (OCT; 10^-7^ M) and pasireotide (PAS; 10^-7^ M) compared to control-treated samples (*n* = 3). (**G**) Fold change of mRNA copy number (measured by qPCR) of apoptotic markers (*CYCS*, *BAX*, *CASP3*, and *CASP8*) in primary GBM cell cultures treated with OCT and PAS compared to control-treated samples (*n* = 5). Data represent means ± SEM. *P < 0.05; **P < 0.01; ****P < 0.0001, significantly different from control conditions. #P < 0.05 significantly different among IDHwt and IDHmt or primary and recurrent samples.


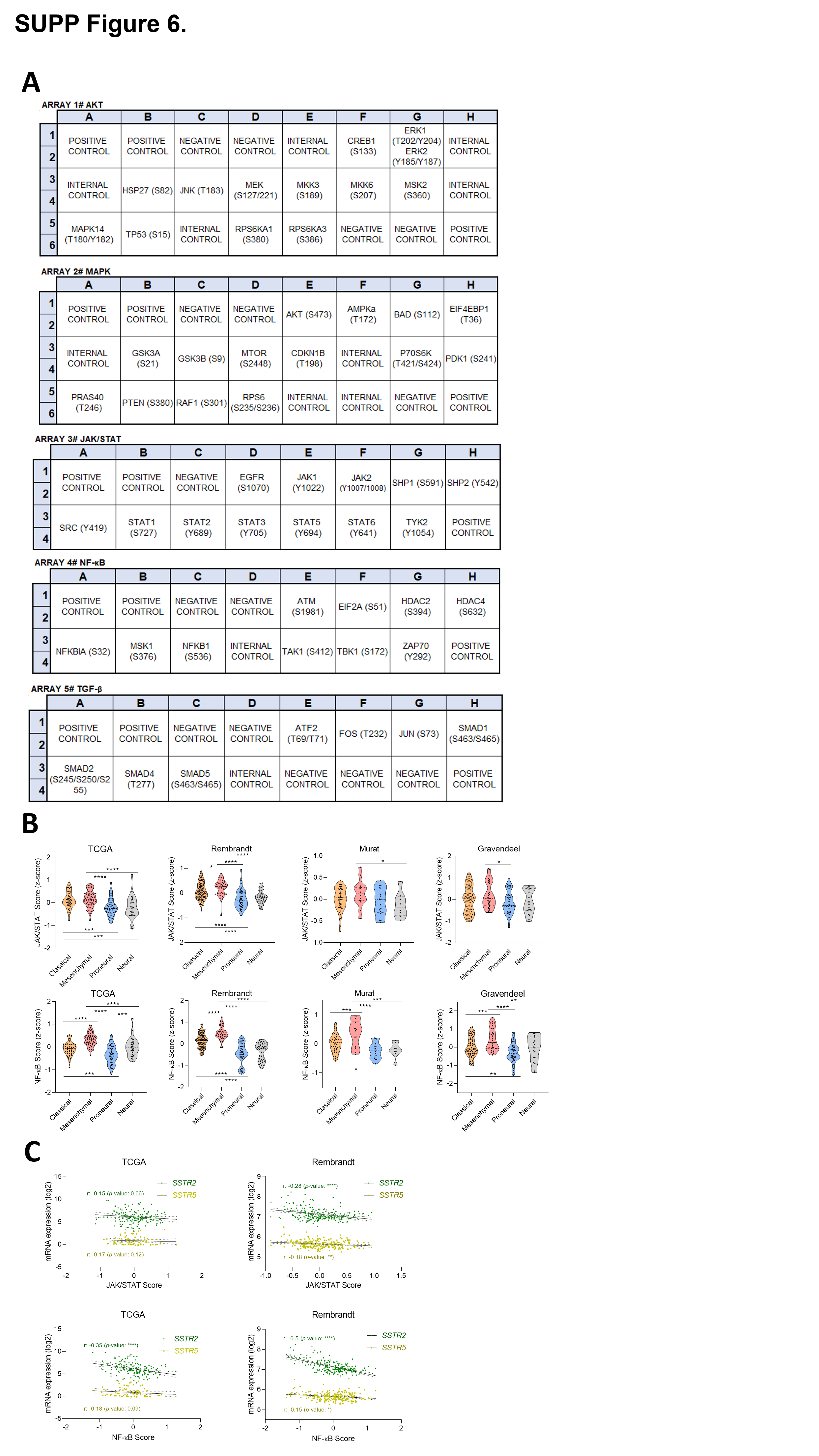


**Fig. S6. Phosphorylation Multi-Pathway Profiling Array Scheme.** (**A**) From top to the bottom, protein targets of AKT pathway, MAPK pathway, JAK/STAT pathway, NF-κB pathway, and TGF-β pathway. POS, Positive Control Spots (controlled amount of detection antibody printed onto the array); NEG, Negative Control Spots (no antibodies); IC, Internal Control spot for RayBiotech. Information on phosphorylated residues is included in parentheses. Each antibody is vertically spotted in duplicate. (**B**) Association between Verhaak subtypes of GBM (Classical, Mesenchymal, Proneural, and Neural) and the JAK/STAT and NF-κB scores from TCGA, Rembrandt, Murat, and Gravendeel datasets. (**C**) correlation plots between SSTR2 (green) and SSTR5 (yellow) expression levels and JAK/STAT and NF-κB scores in the TCGA and Rembrandt external cohorts (r refers to Spearman coefficient). *P < 0.05, **P < 0.01, ***P < 0.001, ****P < 0.0001 significantly different among conditions.

**Supplemental table S1**. Comparison of AUC analyses from SST/CORT-system mRNA expression levels (non-tumour *vs*. GBM samples) obtained in the TCGA external cohort (univariate analysis) **(i)**, and from each individual clinical/molecular feature (TCGA cohort) [Verhaak subtype **(ii)**, *IDH1* status **(iii)**, G-CIMP status **(iv)**, MGMT methylation **(v)**, gender **(vi)**, and age **(vii)**].

| **Feature** | ***SSTR1*** | ***SSTR2*** | ***SSTR3*** | ***SSTR4*** | ***SSTR5*** | ***SST*** | ***CORT*** |
| --- | --- | --- | --- | --- | --- | --- | --- |
| **i)** Univariate AUC analysis | 0.96 / 0.99 | 0.84 / 0.99 | 1 / 1 | 0.98 / 0.99 | 0.7 / 0.61 | 0.98 / 1 | 0.92 / 1 |
| **ii)** Verhaak subtype (classical, mesenchymal, proneural, neural) | 1 / 1 / 0.99 / 1 | 1 / 1 / 0.99 / 1 | 1 / 1 / 1 / 1 | 1 / 1 / 0.98 / 1 | 0.72 / 0.71 / 0.51 / 0.55 | 1 / 1 / 1 / 1 | 1 / 0.95 / 1 / 1 |
| **iii)** *IDH1* (wt/mt) | 1 / 0.97 | 1 / 0.97 | 1 / 1 | 1 / - | 0.64 / 0.75 | 1 / 1 | 1 / 1 |
| **iv)** G-CIMP (positive/negative) | 0.97 / 1 | 0.97 / 1 | 1 / 1 | - / 1 | 0.67 / 0.64 | 1 / 1 | 1 / 1 |
| **v)** MGMT (methylated/  unmethylated) | 1 / 1 | 0.99 / 1 | 1 / 1 | 1 / 1 | 0.59 / 0.57 | 1 / 1 | 1 / 1 |
| **vi)** Gender (female/male) | 1 / 1 | 0.99 / 1 | 1 / 1 | 1 / 1 | 0.65 / 0.58 | 1 / 1 | 1 / 1 |
| **vii)** Age (<50 / > 50) | 0.99 / 1 | 0.99 / 1 | 1 / 1 | 1 / 1 | 0.57 / 0.63 | 1 / 1 | 1 / 1 |
